# Prevalence of upper limb musculoskeletal disorders and its associated risk factors among janitorial workers: A cross-sectional study

**DOI:** 10.1016/j.amsu.2021.103201

**Published:** 2021-12-22

**Authors:** Mei Ching Lim, Khamisah Awang Lukman, Nelbon Giloi, Jac Fang Lim, Richard Avoi, Syed Sharizman Syed Abdul Rahim, Mohammad Saffree Jeffree

**Affiliations:** a Department of Public Health Medicine, Faculty of Medicine & Health Sciences, Universiti Malaysia Sabah, Kota Kinabalu, Sabah, Malaysia; b Centre for Occupational Safety & Health, Universiti Malaysia Sabah, Kota Kinabalu, Sabah, Malaysia

**Keywords:** Awkward postures, Janitorial workers, Job control, Upper limb musculoskeletal disorders, Prevalence

## Abstract

**Introduction:**

Janitorial workers are considered a high-risk group to develop WRMSDs based on their numerous hazardous job tasks and frequent non-fatal injuries being reported. This study aimed to determine the prevalence of upper limb musculoskeletal disorders (ULMSDs) and its associated risk factors among janitorial workers.

**Methods:**

This cross-sectional study involved janitorial workers in a university in Sabah, Malaysia. The participants, who included supervisors, cleaners, and landscape workers, were recruited via universal sampling. Those with at least 12-months of experience in their present employment were included, while those with prior musculoskeletal injuries were excluded. Data were collected through interviews using the Malay version of Standardized Nordic Musculoskeletal Questionnaire (SNMQ), and Job Content Questionnaire (JCQ), followed by Ergonomic Risk Assessment (ERA). Data were analyzed and produced using SPSSv.26, encompassing descriptive statistics, Pearson's Chi-Square, and Multiple Logistic Regression analysis. Ethical approval and respondents' informed consents were obtained prior to the study.

**Results:**

Among 142 respondents, ULMSDs were found to be prevalent in 76.8% of janitorial workers, with the highest prevalence (71.6%) reported in the shoulder regions. None was at negligible risk, with 95.1% in the medium or high-risk categories for RULA assessment. The significant associated factors were landscape workers [aOR = 3.07,95% CI = 1.04, 9.91], more than three years of employment [aOR = 2.47,95% CI = 1.06, 5.79], and low job control [aOR = 2.69,95% CI = 1.16, 6.23].

**Conclusions:**

Given the high prevalence of ULMSDs, risky awkward postures, and low job control, amendments in working apparatuses and improvements in administrative procedures, are highly recommended to prevent the occurrence of ULMSDs.

## Highlights

• WRMSDs is one of the causes of disability globally with significant economic impact.
• The prevalence of WRMSDs among janitorial workers in university setting is scarce.
• Regular screening is essential for monitoring the progress of severity of musculoskeletal symptoms.
• WRMSDs surveillance will be beneficial for improvement of work procedures and practices.

## 1 Introduction

Musculoskeletal disorders (MSDs) involve the nerves, tendons, cartilage, ligaments, joints, and muscles [1]. MSDs are characterized by persistent discomforts, aches, or pain and they frequently cause mobility limitations that restrict the function and productivity of the affected individual [1]. These factors could contribute to an increase in absenteeism, as well as intensifying the demand and cost for medical interventions. MSDs were documented in 1.7 billion people, with majority of them experiencing low back pain that necessitated rehabilitation [2]. Repetitive movements, excessive exertion, long working hours, and poor working environments are common causes of Upper Limb Musculoskeletal Disorders (ULMSDs) [3].

ULMSDs are common among cleaners, especially with their routine manual chores like sweeping, vacuuming, mopping, or scrubbing [4,5]. Cleaning services may require additional heavier tasks like lifting and moving furniture, as well as managing and disposing of waste material [6]. Cleaning work has been linked to a higher incidence of ULMSDs due to the degree of repetitive forced as well as the effects of vibration from cleaning tools [5,7,8]. Furthermore, most cleaners are required to maintain in static or awkward postures, particularly while cleaning higher areas, besides needing to maneuver and balance their bodies in awkward positions when cleaning narrow areas [4,5,8,9].

Aside from that, landscaping work comprises setting up and maintaining plants, trees or lawn; lawn mowing and fertilizing; and tree trimming [10]. Landscaping sector reported high rates of non-fatal injuries, which are mainly caused by overexertion, falls from heights, transportation, exposure to harmful substances or environments [10]. Landscaping tasks include lifting and carrying, stretching, bending over, twisting, pulling, and pushing heavy loads [10,11]. These tasks increase the risk of musculoskeletal disorders, mostly affecting the back, neck, and shoulders [11,12]. Therefore, janitorial workers are undeniably one of the most vulnerable group to develop work-related musculoskeletal disorders (WRMSDs) due to their various hazardous job tasks.

Apart from physical hazards, one of the psychosocial stressors in the janitorial services include the necessity to work alone in isolated settings [4,5]. Workers working alone with no social support, are more susceptible to WRMSDs, as they are expected to handle a wider range of duties. Cleaners are also anticipated to work in a very intense and fast-paced environment, leading to more WRMSDs complaints [13,14]. Long working hours without adequate rest [4,5,13], lack of opinion over work arrangements [4,13], and job seniority [15], are potential psychosocial risk factors for WRMSDs. Besides, janitorial work involved low-skilled jobs with minimal salaries [16]. Hence, the awareness of work-related occupational injuries may be insufficient. Furthermore, with increasing age, the prevalence of MSDs increases as well [17]. Degenerative biological changes in older adults also intensify the likelihood of musculoskeletal injuries among the elderly working population [18].

Occupational, Safety and Health Act (OSHA) emphasized the employer's responsibility in providing as well as maintaining the employee's safety, health, and welfare against any hazards at work [19]. Currently, there is inadequate data on WRMSDs based on job scope, especially among university's janitorial workers. Therefore, this study aimed to determine the prevalence of ULMSDs and its associated risk factors among university's janitorial workers.

## 2 Materials and methods

### 2.1 Methods

This was a cross-sectional study carried out in a public university in Kota Kinabalu, Sabah, Malaysia. The study is conducted in accordance with the declaration of Helsinki [The study was registered in the research registry with Unique Identifying number 7250, and the link is https://www.researchregistry.com/register-now#user-researchregistry/registerresearchdetails/6165788c738d2a0020149863/] and reported according to the STROCSS criteria [20]. Universal sampling involving 166 workers, was used to recruit the participants. Janitorial workers with at least 12-months of working experience in their current job settings were included, while workers who had underlying musculoskeletal injuries were excluded.

Permission was also obtained from the university's Development and Maintenance Department as well as the owners of the janitorial services. The participants were given a thorough overview of the study, including the objectives, methods, benefits, and data confidentiality. Only participants who agreed and signed the consent form were included in the study.

Questionnaires were disseminated to the janitorial workers via their supervisors. Since majority of the workers were illiterate, they were subjected to one-on-one interview with the researcher, assisted by translators who spoke the local dialect. The questionnaires included sociodemographic, past medical history, occupational history, as well Standardized Nordic Musculoskeletal Questionnaire (SNMQ) and Job Content Questionnaire (JCQ).

The validated Malay version SNMQ by Tamrin et al., 2007 [21], which was widely used in various occupations, including bus drivers, manufacturing and plantation workers [14,22,23], was used to evaluate the self-perceived musculoskeletal disorder (MSD) symptoms. It consists of 27 questions with 'No' and 'Yes' answers. MSD symptoms refer to aches, pains, discomfort, or numbness in 9 different body parts over the last 12 months. Besides, assessment of mild to severe symptoms were based on the Canadian Centre for Occupational Health and Safety (CCOHS) [24].

The validated Malay version JCQ was used to assess the psychosocial factors, which consist of psychological job demands, job control, social support, physical demands, and job insecurity [25]. They were evaluated using a four-point Likert scale: 'strongly disagree,' 'disagree,' 'agree,' and 'strongly agree'. The total score in the major scales was calculated using the formula outlined in the Job Content Questionnaire and User Guide [26]. The main scales were further categorized into 'high' and 'low' levels based on the median cut-off point. The reliability of the JCQ, assessed using Cronbach's alpha, was 0.767 for psychological job demand, 0.821 for job control at work, and 0.865 for social support. Hence, the internal consistency was acceptable.

Initial and advanced Ergonomic Risk Assessment (ERA) was conducted following the Malaysian Guidelines on Ergonomics Risk Assessment at Workplace 2017 [27]. A musculoskeletal assessment to identify the various occupational risk factors was conducted, followed by confirmation of the affected body parts using SNMQ. The activities and positions of the workers were then assessed and studied over numerous work cycles to ascertain the ergonomic risk factors and decide whether advanced ERA was required. The postures to be examined were chosen based on following criteria; (i) The most demanding job tasks and postures; (ii) The most constant posture and sustained the longest duration; and (iii) The posture with the highest force loading. Awkward posture, strenuous exertion, and repetitive movements were the preliminary risk factors identified. Scores for initial assessment were given based on the work completed. Factors with scores that met the minimum requirement for the advanced evaluation were followed up with advanced ERA.

The main ergonomic risk identified was awkward postures, and the musculoskeletal symptoms were concentrated in the upper limbs. Hence, Rapid Upper Limb Assessment (RULA) tool, which used a single-page worksheet to assess required body posture, force, and repetition, was chosen for advanced ERA [28]. Scores were entered for each body region in Section A for arm and wrist, and Section B for neck, trunk, and leg based on observations. After the data for each section were collected and scored, tables on the form were used to compile the risk factors variables, generating a single Final RULA grand score, ranging from 1 to 7 and the actions needed to be taken [28]. The pictures and video recordings were analyzed by a certified Ergonomic Trained Personnel to corroborate the researcher's results and avoid study bias.

### 2.2 Statistical analysis

Data were analyzed and produced using SPSS statistical package version 26.0. Descriptive statistics, for instance, frequency, percentage, median and interquartile range, were used to summarize and described the independent variables (sociodemographic characteristics and psychosocial factors) and dependent variable (ULMSDs). Pearson's Chi-Square or Fisher's Exact test (small cell numbers <5) was used to determine the association between categorical independent and dependent variables. Multiple Logistic Regression was used to derive the best-fitting and reasonable model from defining a set of predicted independent variables and its effect on ULMSD. The model will be considered a good fit model based on a few criteria, including Hosmer-Lemeshow goodness-of-fit test and receiver operating characteristic (ROC) curve. All tests were carried out at 5% level of significance.

## 3 Results

Out of 166 workers, only 142 agreed to participate, giving an 88% response rate. Non-respondents were those who were reluctant to participate or request a postponement. The sociodemographic characteristics of janitorial workers are described in Table 1. The age distribution of the workers in this study ranged from 19 to 72 years old. They were divided into two groups based on the median age of 36 (IQR = 19). The majority of the respondents were female (66.2%), from the Bajau ethnic group (78.0%), and married (66.2%). Eighty percent of the respondents had no formal education background or with minimal education level. Landscape workers were mostly paid daily wages, while cleaners and supervisors had fixed monthly incomes. Hence, the range of household income varied from RM400 per month to RM7000 per month. They were categorized into two groups based on the median household income of RM950 (IQR = 150). No significant association of ULMSDs with sociodemographic factors was noted.

**Table 1:** Association of ULMSDs with sociodemographic factors, RULA risk level and psychosocial factors (N = 142).

Risk Factors		ULMSDs			Median (IQR)		df		ꭕ2	p-value
		Yes, n (%)	No, n (%)							
Sociodemographic Factors										
Gender								1	0.235	0.628
Male	38 (79.2)			10 (20.8)						
Female	71 (75.5)			23 (24.5)						
Age Group (years)						36 (19)		1	0.867	0.352
≤36	56 (73.7)			20 (26.3)						
>36	53 (80.3)			13 (19.7)						
Ethnicity								2	1.597	0.450
Bajau	83 (74.8)			28 (25.2)						
Dusun	10 (90.9)			1 (9.1)						
Others	16 (80.0)			4 (20.0)						
Level of Education								1	0.064	0.800
None and Primary School	87 (77.0)			26 (23.0)						
Secondary School and above	22 (75.9)			7 (24.1)						
Household Income						950 (150)		1	1.863	0.172
≤ RM950	61 (81.3)			14 (18.7)						
> RM950	48 (71.6)			19 (28.4)						
Type of Job								1	3.804	0.051
Cleaners	62 (71.3)			25 (28.7)						
Landscape workers	47 (85.5)			8 (14.5)						
Years of Working								1	3.198	0.074
≤ Three years	50 (70.4)			21 (29.6)						
>3 years	59 (83.1)			12 (16.9)						
Smoking Status								1	0.135	0.713
Smoker	20 (74.1)			7 (25.9)						
Non-Smoker	89 (77.4)			26 (22.6)						
**RULA Risk Level**								1	0.204	0.764
Low and Medium Risk	96 (76.2)			30 (23.8)						
Very High Risk	13 (81.3)			3 (18.7)						
**Psychosocial Factors**								1	4.547	0.033^a^
Job Control at Work										
Low	69 (83.1)			14 (16.9)						
High	40 (67.8)			19 (32.2)						
Job Insecurity								1	0.066	0.798
Low	72 (77.4)			21 (22.6)						
High	37 (75.5)			12 (24.5)						
Psychological Job Demand								1	0.270	0.603
Low	65 (78.3)			18 (21.7)						
High	44 (74.6)			15 (25.4)						
Social Support								1	0.721	0.396
Low	62 (79.5)			16 (20.5)						
High	47 (73.4)			17 (26.6)						

IQR = interquartile range.

a Statistically significant if p-value< 0.05.

Based on SNMQ, the overall prevalence of self-reported ULMSDs over 12 months based was 76.8%, involving 109 respondents, while the remaining 33 participants (23.2%) reporting no MSD symptoms. Male workers were noted to have a higher prevalence of self-reported ULMSDs (79.2%). The proportion of ULMSDs was higher among landscape workers (85.5%). The highest prevalence of ULMSDs according to upper body segments was in the shoulders (71.6%), followed by the neck (35.8%), wrist or hands (30.3%), and elbows (4.6%) (Table 2). Nevertheless, MSD symptoms over the neck region was the highest (17.4%) in preventing them from doing their daily work or activities over the past 12 months.

**Table 2:** Prevalence of ULMSDs according to upper body segment (N = 109).

Body Regions	Any trouble^a^ in the last 12 months, n (%)	Prevented from doing regular work or activities, n (%)	Trouble^a^ in the last seven days, n (%)
Neck	39 (35.8)	19 (17.4)	12 (11.0)
Shoulders	78 (71.6)	16 (14.7)	40 (36.7)
Elbows	5 (4.6)	2 (1.8)	2 (1.8)
Wrists/Hands	33 (30.3)	4 (3.7)	8 (7.3)

a Trouble was defined as having symptoms such as ache, pain, discomfort, and numbness.

Workers with self-reported ULMSDs were then further assessed on the severity of their symptoms [24]. Only mild or moderate symptoms were reported. 56.5% of cleaners, comprised of majority female workers, complained of moderate symptoms, while 61.7% of landscape workers who were mostly male, perceived their symptoms were only mild (Table 3). There was a significant association of severity of symptoms with years of working (ꭕ^2^ = 32.30,p < 0.001) and also prevention from work or normal activities (ꭕ^2^ = 5.580,p = 0.018). 74.1% of workers with more than three years of working experience perceived their ULMSDs symptoms were moderate, while 67.9% of workers who needed to rest from their regular work were experiencing moderate symptoms as well.

**Table 3:** Association of perceived severity of symptoms among those with ULMSDs and work-related factors.

Work-related Factors	Severity of Symptoms		df	ꭕ^2^	p-value
	Mild, n (%)	Moderate, n (%)			
Types of Job			1	3.527	0.060
Cleaners	27 (43.5)	35 (56.5)			
Landscape workers	29 (61.7)	18 (38.3)			
Years of Working			1	32.302	p < 0.001^a^
≤ Three years	41 (80.4)	10 (19.6)			
>3 years	15 (25.9)	43 (74.1)			
Prevention from Work or Normal Activities			1	5.580	0.018^a^
Yes	9 (32.1)	19 (67.9)			
No	47 (58.0)	34 (42.0)			
Job Control at Work			1	0.332	0.564
Low	34 (49.3)	35 (50.7)			
High	22 (55.0)	18 (45.0)			
Job Insecurity			1	0.000	0.997
Low	37 (51.4)	35 (48.6)			
High	19 (51.4)	18 (48.6)			
Psychological Job Demand			1	0.024	0.878
Low	33 (50.8)	32 (49.2)			
High	23 (52.3)	21 (47.7)			
Social Support			1	0.690	0.406
Low	34 (54.8)	28 (45.2)			
High	22 (46.8)	25 (53.2)			

a Significant if p < 0.05, N = 109.

In Table 4, none of the workers was in suitable postures while working. 83.8% were in the Medium Risk category, while 11.3% were in the Very High-Risk group, which required immediate intervention to reduce the vulnerability to ULMSDs. As the risk level for awkward posture based on RULA increased, the self-reported prevalence of ULMSDs also increased. Prevalence of ULMSDs of 76.2% in the low- and medium-risk groups increase to 81.3% in the very high-risk group. Fisher's Exact Test analysis revealed no significant association between the prevalence of ULMSDs and RULA risk levels as majority of the janitorial workers were exposed to medium to high levels of ergonomic risk in their daily work chores (Table 1). There was a significant association between job control and ULMSDs among the janitorial workers (p = 0.033). Workers with low job control were more likely to have ULMSDs than those with higher control over their work tasks. However, there was no significant association between other psychosocial factors (job insecurity, psychological job demand, and social support) and ULMSDs (Table 1).

**Table 4:** Distribution of rapid upper limb assessment (RULA) score and risk level among janitorial workers.

Final RULA Score	Level of Risk	Frequency, n	Percentage (%)
3–4	Low Risk	7	4.9
5–6	Medium Risk	119	83.8
7	Very High Risk	16	11.3

Multiple logistic regression was used to determine the predicted independent variables and to what extent these variables affect ULMSDs (the dependent variable). The independent variables chosen were based on the chi-square analysis with p-values < 0.25 from Table 1. In addition, although the RULA risk level had a p-value >0.25, it was included in the multiple logistic regression via enter method because awkward posture, which is assessed by RULA scoring, is a known risk factor for ULMSDs [8,9,17]. Table 5 displays the best final model with no interaction or multicollinearity between the variables noted. Goodness-of-fit model using the Hosmer and Lemeshow test revealed that the model was an excellent fit to the outcome of the data (p > 0.05) and had a good accuracy of 78.9% in predicting the outcome with a sensitivity of 95.5%. ROC curve was noted to be at 0.7, which was reasonably acceptable. Based on multiple logistic regression, landscape workers had three times greater odds of experiencing ULMSDs than cleaners. Janitorial workers who had more than three years working experiences and those with low job control at work were 2.5 and 2.7 times more likely to experience ULMSDs, respectively.

**Table 5:** Multiple logistic regression analysis for predictors of upper limb musculoskeletal disorders.

Variables	B	S.E.	p-value	Adjusted OR	95% CI
Job Category	1.123	0.554	0.043^a^	3.073	1.039; 9.905
Cleaners					
Landscape workers					
Years of Working	0.905	0.434	0.037^a^	2.472	1.056; 5.787
≤ Three years					
>3 years					
Job Control at Work	0.988	0.429	0.021^a^	2.686	1.158; 6.231
High					
Low					
RULA Risk Level	−0.436	0.839	0.604	0.647	0.125; 3.349
Low and Medium Risk					
Very High Risk					
Constant	−0.061	0.395	0.876	0.940	

B = beta coefficient, S. E. = standard error, OR = odds ratio, CI = confidence interval.

a Statistically significant if p-value <0.05.

## 4 Discussion

The overall prevalence of ULMSDs was 76.8%, with workers experiencing discomfort, aches, stiffness, or pain, especially after completing their job tasks. The body region most affected was the shoulders (71.6%), followed by the neck region (35.8%), wrists or hands (30.3%), and elbows (4.6%). Out of the 109 respondents who self-reported ULMSDS, only 31 people (28.4%) reported two or more upper body regions were affected. The result was almost consistent with the previous study conducted among university workers, which reported the highest prevalence of ULMSDs (78.6%) among cleaners [17]. Other researches which involved cleaners, also reported an overall ULMSD prevalence of 78–90% [7,29]. One of the main contributors to the high prevalence of ULMSDs among janitorial workers was because they were all exposed to continuous demanding manual physical work during their 8 h of work daily.

Janitorial workers in this study comprised both the cleaners and landscape workers. Their job tasks required them to have almost similar exposure to awkward posture, repetitive movements, and forceful exertion. Although no significant association was established between gender and ULMSDs, male workers have a higher prevalence of self-reported ULMSDs(79.2%). This finding was inconsistent with other researches [30,31] that indicated a higher prevalence of MSDs among females than males in various working populations. Prevalence of ULMSDs were higher among male workers in this study, mainly because 70.8% of the males were landscape workers who were exposed to more strenuous work chores. Landscape workers were indeed three times more likely than cleaners to experience ULMSDs (aOR = 3.07,95% CI = 1.04, 9.91).

In addition, the prevalence of ULMSDs was higher in the older age group of more than 36 years old (80.3%). This finding was in line with earlier related studies [4,17], that highlighted the prevalence of MSDs increased as age increased. These studies were supported by theories that elderly working population are more vulnerable to musculoskeletal injuries due to degenerative biological changes in older adults, in addition to ongoing persistent imbalance between high physical job demand but low physical working capacity [18].

Even though higher education levels have a slightly lower prevalence of ULMSDs(75.9%) than those with lower education group (77.0%), no significant association was noted between ULMSDs and education level, which also supported the findings from previous studies [14,17]. The prevalence of ULMSDs was reported to be higher among those with lower household income, which are similar to study among Korean workers by Choi et al. (2013) [32]. Workers who were dependent on daily wages, may choose not to rehabilitate even while suffering from mild MSD symptoms due to financial restrictions.

The range of years of working in the same job is from 1 to 26 years. The workers were categorized into two groups based on the median of 3 years of duration of employment. Although there was no significant association between years of working and ULMSDs, workers who had been working for more than three years were noted to have a higher self-reported prevalence of ULMSDs of 83.1%. Janitorial workers who had worked more than three years in the same job scope were persistently exposed to the same risk factors. They were 2.5times more likely to experience ULMSDs (aOR = 2.47,95% CI = 1.06,5.79). The findings from this study concurred with previous studies among sewers and rubber tappers [33,34]. Besides, a significant association was noted between severity of MSDs symptoms with years of working among the janitorial workers (ꭕ^2^ = 32.302, p < 0.001), which was in line with the study conducted by Candan et al.(2019) [35].

There was a significant association between perceived severity of symptoms among those with self-reported ULMSDs and prevention from work or normal activities (ꭕ^2^ = 5.580, p < 0.05). Among those who reported a history of absenteeism from work, 67.9% of them were experiencing moderate MSDs symptoms. The finding was consistent with a previous study conducted among hospital workers by Qhomane-Mhlanga (2014), which reported a significant association between discomfort in affected body regions and absenteeism [36].

The prevalence of ULMSDs increased as the RULA Risk Level increased, from 76.2% in the Low and Medium Risk Level group to 81.3% in the Very High-Risk Level group. None of the workers were in appropriate postures while working. The findings in this study were in line with previous studies in which workers performing manual work experienced more awkward postures [37,38]. No significant association were noted from this analysis.

Janitorial workers were persistently exposed to high physical demands at work. They need to use substantial physical effort, repeating similar tasks and work for long hours. Most cleaners were compelled to maintain in static or awkward postures. The tools they used were inappropriate for their height or petite stature. They occasionally encountered challenging scenarios involving their upper limbs, such as cleaning in narrow areas. Landscape workers were also exposed to awkward posture while performing landscape activities, notably while maneuvering themselves inside narrow drains or balancing their bodies while trimming the hedges or trees, particularly at hilly slopes.

There was a significant association of job control at work with ULMSDs (ꭕ^2^ = 4.547, p < 0.05), which supported the results of previous studies [17,39]. Workers with low job control had 2.7 times higher odds of experiencing ULMSDs (aOR = 2.69,95% CI = 1.16, 6.23). Prevalence of self-reported ULMSDs was higher in the group with low social support (79.5%), as most of them need to work alone, covering a few job tasks. This finding was in line with other studies among university and administrative workers [17,39]. Most of the workers were assured of job sustainability; hence, only 34.5% were noted to have a high level of job insecurity. There was no association between job insecurity and ULMSDs, consistent with the previous study by Amin et al. (2014) [40]. However, previous research Nasaruddin et al. (2014) [37], reported that respondents with high job insecurity were three times more likely than those with low job insecurity to report MSDs. There was no significant association between ULMSDs and psychological job demand, which was consistent with the study done by Burgel et al.(2010) [29].

Few limitations were noted in this study. Data were only collected through interviews followed by observational assessments of their work tasks. No formal medical assessment or report were obtained in this study. As a result, the responses were subjective to recall bias and dependent on the participants' perceptions. Follow up study using medical devices (electromyography or nerve conduction studies) is suggested so that a proper medical diagnosis can be given and progression of symptoms can be monitored. One of the strengths in this study was, ERAs were also conducted to determine the factors that posed the greatest risk to the workers, and the level of risk they faced while completing their daily tasks.

The framework of this study can be utilized to conduct regular MSDs symptoms screening for workers in order to strengthen the WRMSDs surveillance. It will be beneficial in the development of a structured and sustainable program for occupational-related illnesses which include preventive and screening program as well as encourage early notification, intervention, and rehabilitation. This will eventually minimize the labor shortage caused by absenteeism or early retirement owing to WRMSDs.

## 5 Conclusion

High prevalence of ULMSDs (76.8%) were noted among the university's janitorial workers. None of the working postures was suitable, with majority in the medium or high-risk level. The associated risk factors of ULMSDs were landscape workers, more than three years of work experiences, and low job control. The outcome of this study provides valuable information for implementation of control and preventative measures which include powered operated cleaning and landscaping apparatus; mechanical assistances for shifting heavy objects/loads; hands-on-training on ergonomics at work; re-organize work tasks rotation; sufficient resting time in between work and regular open dialogues with supervisor to discuss any impending issues.

## Ethical approval

Ethical clearance from Ethical Committee of Faculty of Medicine and Health Sciences, Universiti Malaysia Sabah (UMS) were obtained before the study was conducted [Approval Code: JKEtika 1/20 (3)]

## Funding

The study was supported by 10.13039/501100006242Universiti Malaysia Sabah under UMSGreat Grant (Project Code: GUG0472-1/2020).

## Author contribution

MCL was involved in the study design, carried out the data collection, conducted the ergonomic risk assessment, performed the statistical analysis and drafted the manuscript. KAL supervised the study design, reviewed the analysis and facilitated the manuscript writing. NG and JFL reviewed the findings of the ergonomic risk assessment. RA reviewed the statistical analysis. MSJ and SSSAR facilitated in writing the manuscript. All authors agreed and approved the final version for publication.

## Consent

Permissions were also granted by the Development and Maintenance Department of UMS as well as from the owners of the janitorial services to conduct the study. All the necessary information was explained to the participants thoroughly which included the nature and purpose of the study, the methods and tools which were used as well as the potential benefits of their involvement in this study. Consent forms were given to the respondents before the study. Only respondents who agreed and signed the consent form were included in the study.

## Registration of research studies

1. Name of the registry: **researchregistry**
2. Unique Identifying number or registration ID: **researchregistry7250**
3. Hyperlink to your specific registration (must be publicly accessible and will be checked): https://www.researchregistry.com/register-now#user-researchregistry/registerresearchdetails/6165788c738d2a0020149863/

## Guarantor

Khamisah Awang Lukman, Department of Public Health Medicine, Faculty of Medicine and Health Sciences, Universiti Malaysia Sabah, Kota Kinabalu, Sabah, MALAYSIA. Email: khamisah@ums.edu.my.

## Declaration of competing interest

The authors report no conflict of interest in this study.
